# TGFβ1-induced SMAD2/3 and SMAD1/5 phosphorylation are both ALK5-kinase-dependent in primary chondrocytes and mediated by TAK1 kinase activity

**DOI:** 10.1186/s13075-017-1302-4

**Published:** 2017-05-31

**Authors:** Arjan van Caam, Wojciech Madej, Amaya Garcia de Vinuesa, Marie-José Goumans, Peter ten Dijke, Esmeralda Blaney Davidson, Peter van der Kraan

**Affiliations:** 1 Experimental Rheumatology, Radboud University Medical Center, Nijmegen, The Netherlands; 2 Orthopaedics Research Lab, Radboud University Medical Center, Nijmegen, The Netherlands; 3 Department of Molecular Cell Biology and Cancer Genomics Centre Netherlands, Leiden University Medical Center, Leiden, The Netherlands

**Keywords:** TGFβ, Smad, Small molecule inhibitor, ALK1, ALK5, TAK1

## Abstract

**Background:**

Dysregulated transforming growth factor β (TGFβ) signaling is implicated in osteoarthritis development, making normalizing TGFβ signaling a possible therapy. Theoretically, this can be achieved with small molecule inhibitors specifically targeting the various TGFβ receptors and downstream mediators. In this study we explore in primary chondrocytes the use of small molecule inhibitors to target TGFβ-induced pSmad1/5/9-, pSmad2/3- and TGFβ-activated kinase 1 (TAK1)-dependent signaling.

**Method:**

Primary bovine chondrocytes and explants were isolated from metacarpophalangeal joints. To modulate TGFβ signaling the activin receptor-like kinase (ALK)1/2/3/6 inhibitor LDN-193189, the ALK4/5/7 inhibitor SB-505124 and the TAK1 inhibitor (5Z)-7-Oxozeaenol were used. pSmad1/5 and pSmad2 were measured using western blot analysis and TGFβ1-induced gene expression was measured using quantitative real time PCR (qPCR).

**Results:**

In chondrocytes, TGFβ1 strongly induced both pSmad1/5 and pSmad2. Remarkably, LDN-193189 did not inhibit TGFβ-induced pSmad1/5. In contrast, SB-505124 did inhibit both TGFβ-induced Smad2 and Smad1/5 phosphorylation. Furthermore, (5Z)-7-Oxozeaenol also profoundly inhibited TGFβ-induced pSmad2 and pSmad1/5. Importantly, both SB-505124 and (5Z)-7-Oxozeaenol did not significantly inhibit constitutively active ALK1, making an off-target effect unlikely. Additionally, LDN-193189 was able to potently inhibit BMP2/7/9-induced pSmad1/5, showing its functionality. On gene expression, LDN-193189 did not affect TGFβ1-induced regulation, whereas both SB-505124 and (5Z)-7-Oxozeaenol did. Similar results were obtained in cartilage explants, although pSmad1/5 was not strongly induced by addition of TGFβ1.

**Conclusion:**

Our data suggest that ALK5 kinase activity plays a central role in both TGFβ-induced Smad1/5 and Smad2/3 phosphorylation, making it difficult to separate both pathways with the use of currently available small molecule inhibitors. Furthermore, our data regarding (5Z)-7-Oxozeaenol suggest that TAK1 facilitates Smad-dependent signaling.

**Electronic supplementary material:**

The online version of this article (doi:10.1186/s13075-017-1302-4) contains supplementary material, which is available to authorized users.

## Background

Transforming growth factor β (TGFβ) plays a crucial role in regulation of tissue homeostasis via its control over diverse cellular processes such as proliferation, differentiation and matrix formation. By binding to different receptors of the activin-receptor like kinase (ALK) family, TGFβ induces intracellular carboxy-terminal (C-terminal) phosphorylation of receptor-regulated Smad (R-Smad) proteins. Phosphorylated R-Smads form complexes with the common-mediator Smad; Smad4, and these complexes translocate to the nucleus where they bind DNA to regulate gene transcription via recruitment of transcription factors [1, 2]. Use of different ALKs can result in activation of different R-Smads; activation of ALK5 (TGFβR1) induces phosphorylation of Smad2 and Smad3, whereas activation of an ALK1 (or 2 or 3) complex mediates phosphorylation of Smad1, Smad5 and Smad9 [3, 4]. However, TGFβ signaling is not limited to R-Smads as Smad-independent signaling can occur by activation of e.g. TGFβ-activated kinase 1 (TAK1 or MAP3K7). Smad-independent signaling results in activation of various downstream signaling pathways, including the JUN N-terminal kinase (JNK) and p38 mitogen-activated protein kinase (MAPK) pathways [5].

In many tissues, e.g. in cartilage, phosphorylated Smad2/3 and Smad1/5 have opposing functions [6, 7]. In cartilage, phosphorylated Smad3 guards chondrocyte phenotype against deleterious hypertrophy and production of catabolic enzymes like matrix metalloproteinases (MMPs) [8–10]. In contrast, phosphorylated Smad1/5 is essential for chondrocyte hypertrophy [11, 12], and is associated with expression of matrix metalloproteinase 13 (MMP13), the main cartilage-degrading enzyme [13]. Therefore, balancing TGFβ signaling via Smad2/3 or Smad1/5 is important for chondrocytes to maintain cellular homeostasis and deregulation of this balance has been proposed as a cause of disease, e.g. osteoarthritis (OA) [4, 13]. It would be of great benefit if cellular responses to TGFβ could be directed towards either the Smad1/5 or Smad2/3 pathway; this would enable blockade of deleterious pathways while maintaining beneficial signaling.

Inhibiting Smad phosphorylation can be achieved by blocking the kinase domain of ALK receptors. Small molecules have been developed that specifically inhibit the kinase activity of ALK4, ALK5 and ALK7, the receptors that phosphorylate Smad2 and Smad3, or ALK1, ALK2, ALK3 and ALK6, the receptors that phosphorylate Smad1, Smad5 and Smad9 [14, 15]. In this study we investigated in chondrocytes and cartilage explants the use of SB-505124 [15, 16], an inhibitor of ALK4/5/7 kinase activity and LDN-193189, an inhibitor of the ALK1/2/3/6 kinase activity [14, 16] to study their ability to direct R-Smad signaling and downstream effects. Furthermore, we used (5Z)-7-Oxozeaenol as a selective inhibitor of TAK1 kinase activity to evaluate the importance of TAK1 in TGFβ signaling in chondrocytes [17, 18], because of the ability of TAK1 to induce the JNK and p38 MAPK pathways and MMP production in cartilage [19, 20].

In this study we show that inhibition of ALK4/5/7 kinase activity prevented TGFβ1-induced C-terminal phosphorylation of both Smad2 and Smad1/5. Furthermore, treatment with (5Z)-7-Oxozeaenol, a selective TAK1 kinase inhibitor, attenuated phosphorylation of both Smad2/3 and Smad1/5 and expression of Smad target genes. Use of the ALK1/2/3 inhibitor LDN-193189 did not affect TGFβ1-induced R-Smad phosphorylation or gene expression. In conclusion, our data show that in cartilage the activity of the ALK5 kinase domain is essential for TGFβ1 signaling, whereas ALK1 kinase activity is not. Furthermore, our results obtained with (5Z)-7-Oxozeaenol underline that TAK1 kinase activity is facilitating R-Smad phosphorylation. Our study indicates that TGFβ1-induced Smad1/5 and Smad2/3 phosphorylation cannot be selectively targeted by using the small molecule inhibitors SB-505124 and LDN-193189.

## Methods

### Chondrocyte culture

Primary bovine chondrocytes were isolated from the metacarpophalangeal joint of cows (2–5 years old) obtained from a slaughterhouse within 3 hours post mortem. Chondrocytes were obtained by incubating cartilage slices overnight in Collagenase B (for details see [21]). Chondrocytes were seeded in DMEM/F12 1:1 containing 10% fetal calf serum (Thermo Scientific UK) at a density of 0.5 × 10^5^ cells per cm^2^ in 6-well plates (Greiner Bio-one International, the Netherlands) for protein studies, or in 24-well plates (Bio-one International, the Netherlands) for mRNA experiments. Cells were cultured for 1 week at 37 °C and 5% CO_2_ before the start of the experiments. For explant studies, four 7-mm^2^ explants per condition were pooled in 1 ml of medium. Both monolayer and explant studies were repeated in different donors.

### Adenoviral transfection of primary chondrocytes

Adenoviruses were used to induce expression of constitutively active (ca)ALK1 [4], constitutively active ALK5 [4] and the CAGA_12_-luciferace reporter construct [22] in primary chondrocytes. Cells were rinsed twice with saline, and adenoviruses were added in a multiplicity of infection of 200 for 3 h at 37 °C in a minimal volume of DMEM/F12 1:1. Hereafter, cells were washed twice with saline, and DMEM/F12 1:1 was added. Cells were used 48 h after transfection.

### Inhibition of TGFβ1 signaling

Before stimulation, cells were deprived of serum for 24 h and thereafter stimulated with 1 ng/ml recombinant human TGFβ1 (Biolegend, the Netherlands) for 2 or 24 h. In experiments where inhibitors were used, DMSO was used as vehicle control. To block TAK1 activity, we used (5Z)-7-Oxozeaenol [17] (Tocris Bioscience) in a concentration of 0.5 μM. To inhibit ALK5 kinase, we used SB-505124 [15] (Sigma Aldrich) in a concentration of 5 μM. For inhibition of ALK1 kinase, LDN-193189 [14] (Axon Medchem) was used in a concentration of 0.05 μM. This concentration of LDN-193189 is well above its reported half maximal inhibitory concentration (IC_50_), 0.8 nM for ALK1, but far below its IC_50_ for ALK5 of 350 nM [16, 23]. Cells were pre-incubated with the inhibitors for 1 h prior to addition of TGF-β1. Either 2 or 24 h after addition TGF-β1, medium was removed and TRI-reagent was added for RNA isolation.

### Protein isolation

To isolate proteins from monolayer cultures, cells were lysed on ice using lysis buffer (Cell signaling, USA) containing a protease inhibitor cocktail (complete, Roche Diagnostics, Germany) and subsequently sonicated on ice using a Bioruptor (Diagenode, USA). With a BCA-assay (Thermo Scientific, USA) protein concentration was measured and after addition of Laemmli sample buffer, samples were boiled for 5 minutes at 99 °C.

To isolate proteins from explants, four 7-mm^2^ explants were homogenized using a Mikro-dismembrator (B. Braun, Germany) and dissolved in 1 ml ice cold radioimmunoprecipitation assay (RIPA) buffer with added 1 mM Na_3_VO_4_ and protease inhibitor cocktail (Roche Diagnostics, Germany). After 1 h incubation on a roller bench at 4 °C, samples were spun down for 3 minutes at 10^4^ × *g* and the pellet was discarded. Cetylpyridinium chloride was added up to a concentration of 1% (m/v) and samples were incubated on a roller bench for 1 h at 4 °C. Hereafter, samples were spun down twice for 15 minutes at 10^4^ × *g* at 4 °C and the pellet was discarded. Using 10 kDa centrifugal filter units (Millipore, USA) the supernatant was concentrated to a volume of 50 μl. Subsequently, proteins were precipitated by addition of 950 μl of 20% m/v trichloroacetic acid and 0.1% m/v dithiothreitol in aceton at -20 °C and spinning samples down for 15 minutes at 6700 × *g* at 4 °C. Next, the pellets were washed three times with 0.1% m/v dithiothreitol in aceton at -20 °C. Finally, the pellets were dried under vacuum for 20 minutes and dissolved in 100 μl 1% m/v sodium dodecyl sulfate in 100 mM tris(hydroxymethyl)aminomethane in H_2_O pH 9.0.

### Detection of proteins using SDS-PAGE and western blot

Proteins were seperated on a 7.5% Bisacrylamide gel, and transferred to a nitrocellulose membrane using wet transfer (Towbin buffer, 2.5 h 275 mA at 4 °C). After overnight incubation at 4 °C with 1:1000 polyclonal Rabbit anti P-Smad1/5 (S463/465)/Smad8 (S426/428) (Cell signaling, USA) or anti P-Smad2 (S465/467) (Cell signaling, USA), membranes were incubated with 1:1500 polyclonal Goat anti Rabbit labeled with horseradish peroxidase (HRP) (DAKO, Belgium) for 1 h. Hereafter, enhanced chemiluminescence using ECL plus (GE Healthcare, UK) was used to visualize the proteins. To visualize overexpression of constitutively active ALKs, a rabbit polyclonal antibody directed against their internal HA tag was used: HA-probe Antibody (Y-11) (Santa Cruz, USA) (1:1000). As loading control either Gapdh was stained with an anti-Gapdh mouse mAb (1G5) (Sigma Aldrich, Germany) (1:10 000) in combination with IRDye 680RD Donkey anti mouse (1:10 000) (Licor, USA) using the Odyssey detection system (Licor, USA) or Vinculin with a rabbit pAb (H300) (Santa Cruz, USA) (1:1000) in combination with HRP-labeled Goat-anti Rabbit (1:2000) (DAKO, Denmark) using ECL. Finally, blots were quantified using ImageJ.

### Detection of gene expression

TRI-reagent (Sigma-Aldrich, Germany) was used for RNA isolation according to the manufacturer’s protocol (for details see [21]). RNA concentration was measured using a Nanodrop photospectrometer (Thermo Scientific, USA). Per sample, 1 μg of RNA was treated with DNAse (Life Technologies, USA), which was subsequently inactivated at 65 ° C with 1 μl 25 mM EDTA (Life Technologies, USA). To perform reverse transcriptase reaction, 1.9 μl ultra pure water, 2.4 μl 10 × DNAse buffer, 2.0 μl 0.1 M DTT, 0.8 μl 25 mM dNTP, 0.4 μg oligo dT primer, 1 μl 200 U/μl M-MLV Reverse transcriptase (all Life Technologies, USA) and 0.5 μl 40 U/μl RNAsin (Promega, the Netherlands) was added, and samples were incubated for 5 minutes at 25 °C, 60 minutes at 39 °C, and 5 minutes at 95 °C using a thermo cycler. The obtained cDNA was diluted 10 times in ultra pure water, and gene expression was measured using 1 μM of validated cDNA-specific primers (Biolegio, the Netherlands; see Table 1) in a quantitative real-time polymerase chain reaction (qPCR) using SYBR green master mix (Applied Biosystems). The following protocol was used: after 10 minutes at 95 °C, 40 cycles of 15 sec at 95 °C and 1 minute at 60 °C each were run. Hereafter a melting curve was made to verify gene-specific amplification. For calculations of the -ΔCt, two reference genes were used: *glyceraldehyde 3-phosphate dehydrogenase* and *ribosomal protein S14*.

**Table 1:** Sequence and efficiency of the primers used in this study

Gene	NCBI reference sequence	P (bp)	E (%)	Forward primer	Reverse primer
*bGapdh*	NM_001034034.2	90	101	CACCCACGGCAAGTTCAAC	TCTCGCTCCTGGAAGATGGT
*bRps14*	NM_001077830.2	125	105	CATCACTGCCCTCCACATCA	TTCCAATCCGCCCAATCTTCA
*bAlk1*	NM_001083479.1	109	100	ACAACACAGTGCTGCTCAGACA	TGCTCGTGGTAGTGCGTGAT
*bAlk2*	NM_176663.3	61	105	CGTTGGAGACAGCACTTTAGCA	AGAGCCGCTTCCCGATGTA
*bAlk3*	NM_001076800.1	142	96	GAGCAAGATGAAGCATTTATTCCA	CAACCTGCCGAACCATCTG
*bAlk5*	NM_174621.2	75	94	CAGGACCACTGCAATAAAATAGAACTT	TGCCAGTTCAACAGGACCAA
*bSerpine1*	NM_174137.2	55	99	CGAGCCAGGCGGACTTC	TGCGACACGTACAGAAACTCTTGA
*bId1*	NM_001097568.2	73	107	GCTCCGCTCAGCACTCTCAA	GATCGTCCGCTGGAACACA
*bMmp3*	NM_001206637.1	130	106	GAATCTGTGCCTCCCGAACC	TCCTGAAAGATTTCCGCCAA
*bSmad7*	NM_001192865.1	72	98	GGGCTTTCAGATTCCCAACTT	CTCCCAGTATGCCACCACG
*bTgfb1*	NM_001166068.1	80	107	GGTGGAATACGGCAACAAAATCT	GCTCGGACGTGTTGAAGAAC
*bJunb*	NM_001075656.1	139	97	CCTTCTACCACGACGACTCA	CCGGGTGCTTTGAGATTTCG
*bNgf*	NM_001099362.1	110	85	CAGCTCTTTTGATCGGCATACA	TGTGTCAAGGGAATGCTGAAGT
*bFn1*	NM_001163778.1	95	96	GCACCACTCCCGACATTACT	CTGATCGGCATGGACCACTT
*bCol2a1*	NM_001001135.2	60	97	TGATCGAGTACCGGTCACAGAA	CCATGGGTGCAATGTCAATG

P is product length in base pairs, E is efficiency in percentage. *Alk1* is also known as *Acvrl1*, *Alk2* as *Acvr1*, *Alk3* as *Bmpr1a* and *Alk5* as *Tgfbr1*

### Statistics

All quantitative data are expressed as a mean of multiple repeats ± SD. For every analysis data were checked for normality using the Shapiro-Wilk test. One-way analysis of variance (ANOVA) with Tukey multiple comparison post-hoc test was used to determine the significance. The statistical analyses were performed using Graphpad Prism 5.0 software.

## Results

### SB-505124 and (5Z)-7-Oxozeaenol both inhibit TGFβ1-induced Smad1/5 and Smad2/3 phosphorylation whereas LDN-193189 does not

**Fig. 1:**
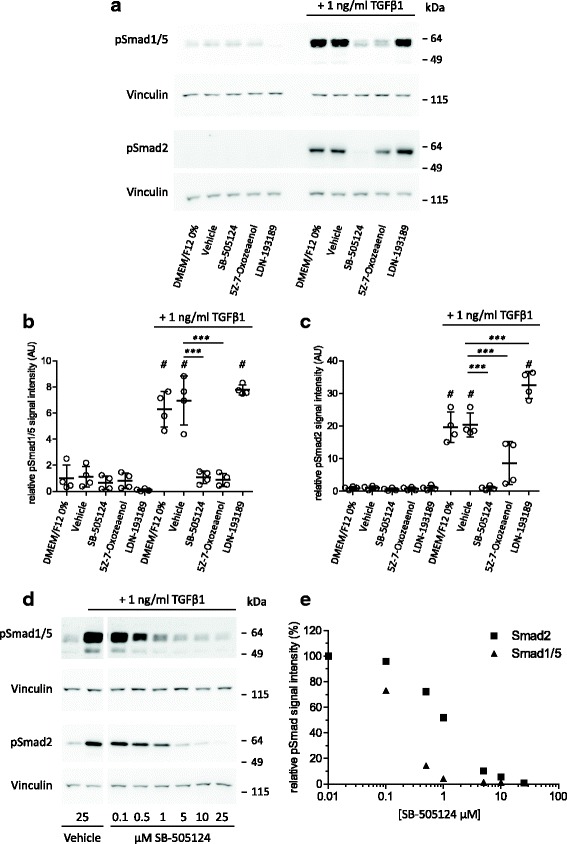
SB-505124 and (5Z)-7-Oxozeaenol inhibit both transforming growth factor β1 (*TGFβ1*)-induced Smad1/5 and Smad2/3 phosphorylation whereas LDN-193189 does not. **a** Primary bovine chondrocytes were pre-incubated with either 5 μM SB-505124, 0.5 μM (5Z)-7-Oxozeaenol or 0.05 μM LDN-193189 for 1 h, and subsequently stimulated with 1 ng/ml TGFβ1 for 1 h, after which phosphorylated Smads were visualized using western blot by specific antibodies. kDa, kilodalton. **b** Quantification of the pSmad1/5 signal (as shown in **a**) in four experiments. pSmad1/5 levels were normalized to vinculin levels and plotted as a relative amount in arbitrary units (*AU*) compared to the control group: #*p* ≤ 0.001 compared to unstimulated; ***p ≤ 0.001. **c** Quantification of the pSmad2 signal (as shown in **a**) in four experiments. pSmad2 levels were normalized to vinculin levels and plotted as a relative amount in AU compared to the control group: ^#^ *p* ≤ 0.001 compared to unstimulated; ***p ≤ 0.001. **d** Dose-response effect of SB-505124 on TGFβ1 signaling in primary chondrocytes. **e** Quantification of pSmad levels (shown in **d**); levels were normalized to vinculin levels and plotted as a relative amount in percentage compared to the control group

To begin, we confirmed in primary bovine chondrocytes the expression of the receptors that TGFβ can use to induce Smad2/3 phosphorylation i.e. ALK5 or Smad1/5 phosphorylation, i.e*.* ALK1, ALK2 and ALK3 [3, 4]. All four receptors were readily detected using qPCR (Additional file 1: Figure S1), and indeed confirmed that stimulation of chondrocytes with TGFβ1 results in both Smad2 and Smad1/5 phosphorylation (Fig. 1a).

Subsequently, we analyzed the effects of the ALK4/5/7-kinase inhibitor SB-505124, the ALK1/2/3-kinase inhibitor LDN-193189 and the TAK1-kinase inhibitor (5Z)-7-Oxozeaenol on TGFβ1-induced R-Smad phosphorylation (Fig. 1a, b and c). Strikingly, addition of 5 μM SB-505124 not only inhibited Smad2 phosphorylation, but also pSmad1/5 phosphorylation. Remarkably, treatment with 0.5 μM (5Z)-7-Oxozeaenol also resulted in inhibition of TGFβ1-induced Smad1/5 and Smad2 phosphorylation. Noteworthy, addition of 0.05 μM LDN-193189 did not affect TGFβ1-induced Smad1/5 phosphorylation but did significantly enhance TGFβ-induced pSmad2.

**Fig. 2:**
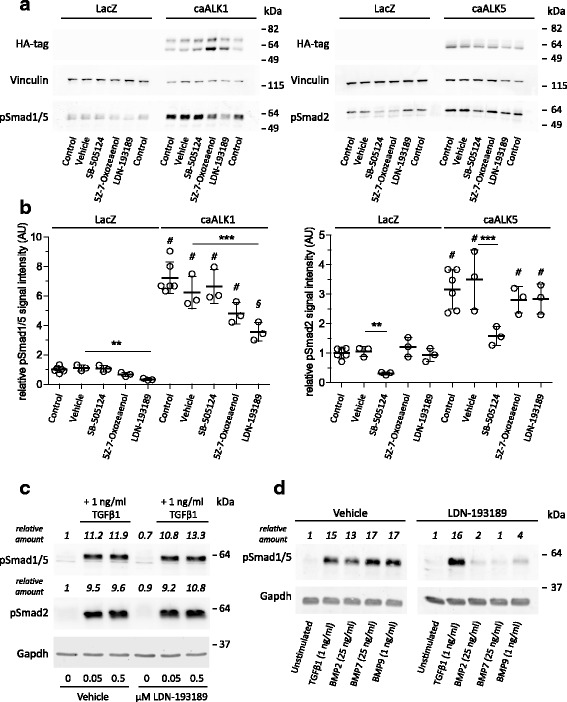
SB-505124 and (5Z)-7-Oxozeaenol do not affect constitutively active activin receptor-like kinase 1 (*ALK1*) whereas LDN-193189 does. **a** Primary bovine chondrocytes were transfected with either constitutively active ALK1 (*caALK1*) or caALK5 and 2 days later treated with inhibitors for 1 h. A virus overexpressing LacZ was used as control. Efficiency of overexpression was visualized on western blot by staining for the HA-tag attached to the caALKs. *kDa* kilodalton. **b** Quantification of the pSmad1/5 and pSmad2 signal (as shown in **a**) in three experiments; ^#^ *p* ≤ 0.001 compared to unstimulated. pSmad levels were normalized to vinculin levels and plotted as a relative amount in arbitrary units (*AU*) compared to the control group; ^§^ *p* ≤ 0.01 compared to unstimulated; **p ≤ 0.01; ***p ≤ 0.001. **c** Dose-response effect of LDN-193189 on transforming growth factor β1 (*TGFβ1*) signaling in primary chondrocytes. pSmad levels were normalized to glyceraldehyde-3-phosphate dehydrogenase (*Gapdh*) levels and indicated as a relative amount in AU compared to the control group. **d** Primary chondrocytes were incubated for 1 h with LDN-193189 and subsequently stimulated for 1 h with 1 ng/ml (78 pM) TGFβ1, 25 ng/ml (1.94 nM) recombinant human bone morphogenic protein 2 (*rhBMP2*), 25 ng/ml (3.06 nM) bone morphogenic protein 7 (*BMP7*) or 1 ng/ml (83 pM) bone morphogenic protein 9 (*BMP9*). pSmad levels were normalized to Gapdh levels and indicated as a relative amount in AU compared to the control group

To investigate if the striking effect of SB-505124 on Smad1/5 phosphorylation was a dose-dependent effect, we performed a dose-response experiment (Fig. 1d and e). This experiment showed that at all tested doses (0.1, 0.5, 1, 5, 10 and 25 μM), SB-505124 inhibited TGFβ1-induced Smad1/5 phosphorylation, and that Smad1/5 phosphorylation was inhibited more potently than that of Smad2. Therefore, to address if ALK1 inhibition was a possible off-target effect of SB-505124, this compound was added to cells over-expressing constitutively active (ca)ALK1 or caALK5. This experiment showed that SB-505124 did not affect caALK1-induced Smad1/5 phosphorylation (Fig. 2a and b), but did inhibit caALK5-induced Smad2 phosphorylation (Fig. 2a and b). Therefore, SB-505124 is specific for ALK5 kinase and does not inhibit ALK1 kinase. The same experimental setup was used to address the specificity of (5Z)-7-Oxozeaenol, and no significant inhibitory effect of this compound on either caALK1 or caALK5 function was detected, making it unlikely that either the ALK1 or ALK5 kinase domain is an off-target of (5Z)-7-Oxozeaenol.

To investigate if the lack of effect of LDN-193189 on TGFβ1-induced Smad1/5 was maybe dose-dependent, we added a 10 times higher dose of LDN-193189 (0.5 μM) to the cells, but were still unable to inhibit TGFβ1-induced Smad1/5 phosphorylation (Fig. 2c). Therefore we sought to validate the function of LDN-193189. To do this, we added 0.05 μM LDN-193189 to chondrocytes over-expressing caALK1 and observed that this blocked caALK1-induced Smad1/5 phosphorylation (Fig. 2a and b), Furthermore, the effect of 0.05 μM LDN-193189 on bone morphogenic protein (BMP) signaling was also investigated (Fig. 2d) by stimulating primary chondrocytes for 1 h with either the ALK2/3 ligands BMP2 or BMP7 [24] or the ALK1/2 ligand BMP9 [25, 26]. We observed that BMP-induced Smad1/5 phosphorylation was clearly inhibited by 0.05 μM LDN-193189. Together, these experiments strongly confirm the function of 0.05 μM LDN-193189 in primary chondrocytes.

In conclusion, in primary chondrocytes, TGFβ1-induced Smad1/5 phosphorylation is unaffected by LDN-193189, whereas both Smad2/3 and Smad1/5 phosphorylation are inhibited by SB-505124 and (5Z)-7-Oxozeaenol.

### SB-505124 fully and (5Z)-7-Oxozeaenol partly inhibit TGFβ1-induced Smad-dependent gene expression whereas LDN-193189 does not

**Fig. 3:**
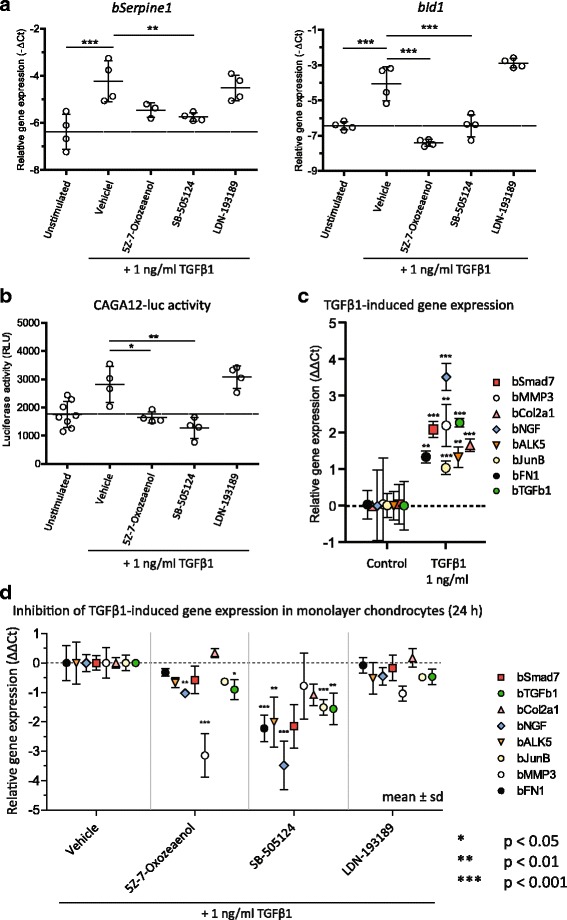
SB-505124 fully and (5Z)-7-Oxozeaenol partly inhibits transforming growth factor β1 (*TGFβ1*)-induced Smad-dependent gene expression whereas LDN-193189 does not. **a** Primary bovine chondrocytes were pre-incubated with 5 μM SB-505124, 0.5 μM (5Z)-7-Oxozeaenol or 0.05 μM LDN-193189 for 1 h, and subsequently stimulated with 1 ng/ml TGFβ1 for 2 h and 24 h. With the use of quantitative (q)PCR, expression of *bSerpine1* was measured as response gene for pSmad3 signaling after 2 h, and *bId1* for pSmad1/5 signaling after 2 h. **b** The pSmad3 responsive CAGA_12_-luciferase (*CAGA*_*12*_ *-luc*) construct was placed in primary chondrocytes with the use of an adenovirus, and cells were stimulated with 1 ng/ml TGFβ1 for 8 h with or without inhibitors after 2 days. **c** Primary chondrocytes were stimulated with 1 ng/ml TGFβ1 for 24 h and gene expression was measured using validated cDNA-specific primers and qPCR. *MMP* matrix metalloproteinase, *NGF* nerve growth factor, *ALK5* activin receptor-like kinase 5. **d** Gene expression was measured after pre-incubation of chondrocytes for 1 h with inhibitors followed by stimulation with 1 ng/ml TGFβ1 for 24 h. For qPCR data, average ± sd (*mean ± sd*) was plotted, with each *dot* representing the average of one donor (**a**) or four donors (**c** and **d**). Analysis was performed using one-way analysis of variance with Tukey’s post-hoc test: *p ≤ 0.05; **p ≤ 0.01; ***p ≤ 0.001. A change of 1 *ΔΔC*_*t*_ equals twofold upregulation

Next, we analyzed the effects of TGFβ1 signaling inhibitors on TGFβ1-induced Smad-dependent gene expression. For this, *bId1* and *bSerpine1* were used as markers of pSmad1/5 and Smad3 signaling, respectively [4, 27] [22, 28]. Expression of *bId1* was profoundly upregulated by stimulation with 1 ng/ml of TGFβ1 (Fig. 3a). In accordance with our western blot data on pSmad1/5, expression of *bId1* was inhibited by both SB-505124 and (5Z)-7-Oxozeaenol, and was unaffected by LDN-193189. Expression of *bSerpine1* was upregulated by 1 ng/ml of TGFβ1 and fully inhibited by SB-505124, but was only partly inibited by (5Z)-7-Oxozeaenol (Fig. 3b). To confirm these effects on pSmad3-dependent gene expression, we used the CAGA_12_-luc construct, which produces luciferase specifically in response to pSmad3. After 8 h, TGFβ-induced CAGA_12_-luc activity was significantly inhibited by (5Z)-7-Oxozeaenol and SB-505124, showing that pSmad3-signaling is indeed lowered in these conditions. When the effect of the inhibitors on multiple TGFβ1-induced (Fig. 3c), genes like *bSmad7*, *bMmp3*, *bCol2a1*, *bNgf*, *bAlk5*, *bJunb*, *bFN1* and *bTgfb1* was measured, none of these genes was significantly downregulated by LDN-193189, unlike SB-505124, which profoundly downregulated *bSmad*, *bNgf*, *bAlk5*, *bJunb*, *bFN1* and *bTgfb1*, or unlike treatment with (5Z)-7-Oxozeaenol, which significantly lowered *bNgf*, *bTgfb1* and *bMmp3* expression (Fig. 3d). Because a role for pSmad1/5 in the regulation of e.g*. Smad7*, *Junb* and *Col2a1* has been established [29–31], these data indicate that the ALK5 kinase inhibitor SB-505124 inhibits gene expression downstream of both pSmad2/3 and pSmad1/5, whereas (5Z)-7-Oxozeaenol does so to a lesser extent and in a more limited amount of genes. No effect of LDN-193189 was observed on TGFβ1-induced gene expression.

### In intact cartilage, SB-505124 and-Oxozeaenol inhibit TGFβ1-induced pSmad1/5 and pSmad2

**Fig. 4:**
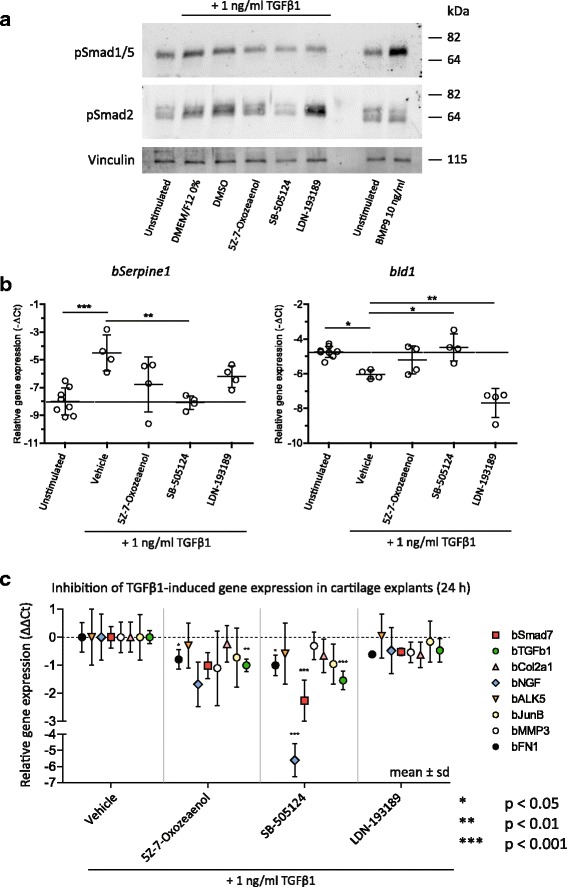
In cartilage, SB-505124 fully inhibits and (5Z)-7-Oxozeaenol partly inhibits transforming growth factor β1 (*TGFβ1*)-induced Smad2/3 phosphorylation and gene expression, whereas LDN-193189 does not. **a** Bovine cartilage explants were pre-incubated ex vivo with 5 μM SB-505124, 0.5 μM (5Z)-7-Oxozeaenol or 0.05 μM LDN-193189 for 1 h, and subsequently stimulated with 1 ng/ml TGFβ1 for 2 h, after which phosphorylated Smads were visualized by western blot using specific antibodies. *kDa* kilodalton. **b** With the use of quantitative (q)PCR, expression of *bSerpine1* was measured as response gene for pSmad3 signaling after 24 h, and *bId1* for pSmad1/5 signaling after 24 h. **c** Gene expression was measured after pre-incubation of explants for 1 h with inhibitors followed by stimulation with 1 ng/ml TGFβ1 for 24 h. For qPCR data, average ± sd (*mean ± sd*) was plotted, with each *dot* representing the average of one donor (**b**) or four donors (**c**). Analysis was performed using one-way analysis of variance with Tukey’s post-hoc test: *p ≤ 0.05; **p ≤ 0.01; ***p ≤ 0.001). A change of 1 *ΔΔC*_*t*_ equals twofold upregulation or downregulation. *TGF* transforming growth factor, COL2A1 = collagen type II alpha 1 chain *MMP* matrix metalloproteinase, *NGF* nerve growth factor, *ALK5* activin receptor-like kinase, JUNB = JunB proto-oncogene, FN1 = fibronectin 1

As TGFβ1 signaling is known to be affected by cellular context, we sought to validate our observations in monolayer chondrocytes in intact cartilage ex vivo. Therefore we stimulated cartilage explants with 1 ng/ml TGFβ1 with or without inhibitors, and investigated Smad phosphorylation. Explants were stimulated for 2 h to allow for diffusion of TGFβ1 through the cartilage matrix. In explants, 1 ng/ml TGFβ1 induced pSmad2, but Smad1/5 phosphorylation was more difficult to detect (Fig. 4a), in contrast to in monolayer cultures. Therefore BMP9 was used as positive control, which, in comparison to TGFβ, resulted in far stronger induction of pSmad1/5 (for quantification see Additional file 2: Figure S3). A smaller amount of pSmad1/5 was detected in explants treated with TGFβ1 and SB-505124, (5Z)-7-Oxozeaenol or LDN-193189 compared to TGFβ1-treated samples alone. TGFβ-induced phosphorylation of Smad2 was clearly inhibited by SB-505124 and partly by (5Z)-7-Oxozeaenol (Fig. 4a), but on gene expression *bSerpine*1 was only significantly inhibited by SB-505124 and not by (5Z)-7-Oxozeaenol (Fig. 4b). In explants, expression of *bId1* could not be used as a read-out for pSmad1/5 signaling, because TGFβ1 downregulated its expression, in contrast to its induction in monolayer experiments. This is a well-known Smad3-dependent cytostatic effect of TGFβ1 [32], and further indicates that TGFβ1 signaling differs between chondrocytes in monolayer and chondrocytes in intact cartilage. Notably, LDN-193189 did downregulate *bId1* expression, even in absence of TGFβ1 (Additional file 3: Figure S2), demonstrating the bioactivity of this compound in cartilage explants. Again, when multiple, TGFβ1-induced, Smad-dependent, genes were measured (Fig. 4c) SB-505124 inhibited TGFβ1-induced expression of *bSmad7*, *bNgf*, *bFn1* and *bTgfb1*, and (5Z)-7-Oxozeaenol inhibited TGFβ1-induced expression of *bFn1*, *bTgfb1,* whereas no effect of LDN-193189 was observed. Therefore, SB-505124 also inhibits both TGFβ1-induced Smad1/5 and Smad2 phosphorylation and gene expression in intact cartilage, whereas LDN-193189 does not. Furthermore, (5Z)-7-Oxozeaenol partly inhibited both TGFβ1-induced Smad1/5 and Smad2 phosphorylation.

## Discussion

TGFβ1 signaling is a crucial regulator of chondrocyte homeostasis. We investigated the impact of small molecule inhibitors on TGFβ1 signaling in primary chondrocytes both in vitro and ex vivo. We report that inhibition of ALK4/5/7 kinase with SB-505124 lowered both TGFβ1-induced Smad1/5 and Smad2/3 phosphorylation and downstream gene expression, whereas inhibition of ALK1/2/3/6 kinase with LDN-193189 did not affect TGFβ1-induced Smad1/5 phosphorylation or transcriptional activity. In addition, we showed that treatment with (5Z)-7-Oxozeaenol, a selective TAK1 kinase inhibitor, attenuated TGFβ1-induced R-Smad phosphorylation and downstream gene expression.

**Fig. 5:**
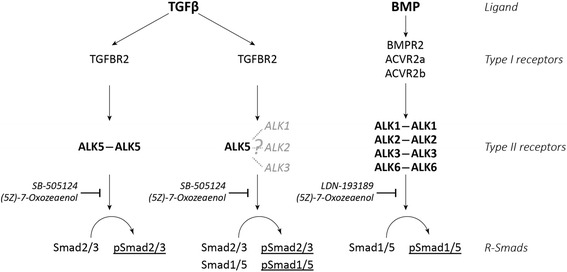
Schematic overview of transforming growth factor β (*TGFβ*)-induced Smad signaling versus bone morphogenic protein (*BMP*)-induced Smad signaling in cartilage and the effects of small molecule inhibitors. To signal, a TGFβ dimer binds and induces dimerization of its type II receptor: *TGFBR2*. This complex subsequently recruits a dimer of the type I receptors TGFβ can bind: activin receptor-like kinase (*ALK*)1, ALK2, ALK3 and ALK5. Signaling complexes containing ALK5 homodimers will induce Smad2/3 phosphorylation, whereas complexes containing ALK5 combined with ALK1, ALK2 or ALK3 will induce both Smad1/5 and Smad2/3 phosphorylation. However, our current study shows that the kinase domain of ALK1, ALK2 or ALK3 is not involved in TGFβ-induced pSmad1/5 because TGFβ-induced pSmad1/5 is inhibited by SB-505124 but not by LDN-193189. Possibly, ALK1, ALK2 and ALK3 function to recruit Smad1/5 to TGFβ receptor complexes but are not activated by TGFBR2 themselves and are therefore not active. For BMP signaling a heterotetrameric complex is also formed from its different type II and type I receptors; *BMPR2*, *ACVR2a* and *ACVR2b* can be used as type II receptors, whereas ALK1, ALK2, ALK3 and ALK6 function as type I receptors. These complexes will induce pSmad1/5 but this is potently blocked by LDN-193189 (as shown in this article) or partially blocked by (5Z)-7-Oxozeaenol (evidence from the literature)

In many tissues, including cartilage, TGFβ1 induces pSmad1/5 and pSmad2/3 [3, 4, 7]. ALK5 is essential for TGFβ1-induced Smad2/3 phosphorylation, whereas a role for ALK1 [4], ALK2 and ALK3 [3] has been demonstrated in TGFβ-induced Smad1/5 phosphorylation (see Fig. 5). Remarkably, we found that specific inhibition of ALK4/5/7 kinase with SB-505124 was sufficient to inhibit both TGFβ1-induced Smad2/3 and Smad1/5 phosphorylation and transcriptional activity. In contrast, we were unable to block TGFβ1-induced Smad1/5 phosphorylation with LDN-193189, even though we used a dose (0.05 μM) well above the reported IC_50_ of this inhibitor for ALK1 (0.8 nM), ALK2 (0.8 nM) and ALK3 (5.3 nM) [23]. Additionally, even a 10 times higher dose of LDN-193189 (0.5 μM) was unable to block TGFβ1-induced Smad1/5 phosphorylation. LDN-193189 has been established as an efficient inhibitor of BMP type I receptors, i.e. ALK1/2/3 and ALK6, both in vitro and in vivo [14, 23, 33]. We were able to confirm this inhibitory effect of LDN-193189 on BMP-induced Smad1/5 phosphorylation in primary chondrocytes by using BMPs that signal via ALK1, ALK2 and/or ALK3, i.e. BMP2, BMP7 and BMP9 [24, 25]. Furthermore, we were also able to block caALK1 activity with LDN-193189 in our primary cell cultures. Together, these experiments indicate that LDN-193189 is functional in primary bovine chondrocytes and blocks the ALK1, ALK2 and ALK3 kinase activity at a dose of 0.05 μM. Therefore, our data indicate that ALK1, ALK2 or ALK3 kinase activity is not required for TGFβ1-induced Smad1/5 in primary chondrocytes and cartilage. Notably, various studies using the LDN-193189 precursor dorsomorphin also show that this BMP type I receptor blocker is unable to block TGFβ1-induced Smad1/5 phosphorylation, supporting our observations [34, 35].

Based on structural analyses of R-Smad-ALK interactions, the ability to phosphorylate Smad1/5 is thought to be limited to BMP type I receptors [36, 37]. Our data indicate that ALK5 kinase activity is crucial, and possibly sufficient, for TGFβ1-induced Smad1/5 phosphorylation. In line with our data, it has previously been demonstrated that inhibition of ALK5-kinase with SB-431542 in endothelial cells [6] or with SB-505124 in synovial fibroblasts [34] attenuates TGFβ1-induced phosphorylation of Smad1/5. Furthermore, by knocking down ALK5, it has been shown by others that this receptor is necessary for TGFβ-induced Smad1/5 phosphorylation [3, 6]. Possibly, ALK5 can directly phosphorylate Smad1/5 independently of BMP type I receptors in cartilage. Direct phosphorylation of Smad1 by caALK5 has been demonstrated by in vitro kinase assays [35, 38], and it has been shown that TGFβ1-induced Smad1 phosphorylation is inhibited in cells expressing a kinase defective ALK5 variant: ALK5KR [38]. Together, these studies indicate a direct induction of pSmad1/5 by ALK5, which would be blocked by SB-505124, but challenge the dogma that ALK5 cannot directly interact with Smad1 [36, 37]. In contrast, multiple studies show that in the absence of BMP type I receptors, TGFβ1-induced pSmad1/5 is reduced [3, 6] indicating at least a role for these receptors.

Apart from R-Smad phosphorylation, TGFβ1 also induces TAK1 activation, a key component of Smad-independent TGFβ signaling [5]. Noteworthy, with the use of the specific TAK1 kinase inhibitor (5Z)-7-Oxozeaenol [17] at 0.5 μM, we showed that TAK1 kinase activity facilitates canonical C-terminal R-Smad phosphorylation in chondrocytes and cartilage. Previous studies support a role for TAK1 in R-Smad-dependent signaling; with the use of cartilage-specific TAK1 knockout animals, it has been shown that loss of TAK1 greatly diminishes BMP-induced C-terminal Smad1/5 phosphorylation while not affecting total R-Smad levels [39, 40]. Furthermore, tissue specific knockout of TAK1 in mesenchymal cells of neural crest origin shows that also C-terminal Smad2/3 phosphorylation is inhibited in the absence of TAK1 [41]. The pronounced effects of TAK1 on canonical C-terminal Smad phosphorylation have been ascribed to TAK1-induced Smad-linker phosphorylation, a post-translational Smad modification [41, 42].

Linker modification of R-Smads is an essential aspect of TGFβ1-signaling (reviewed in [43, 44]). The linker region of R-Smads contains threonine and serine residues that can be phosphorylated by various intracellular kinases like extracellular signal-regulated kinases (ERK), cyclin-depdendent kinases (CDKs) and JNKs [43, 45]. Degradation, nuclear transport and interaction of R-Smads with other proteins are regulated by phosphorylation of this region, enabling context-dependent TGFβ signaling [43, 44]. TAK1 can directly interact with R-Smads via the R-Smad MH2 domain [42], and phosphorylate, e.g. Smad2 at threonine 220 (Thr-220). Phosphorylation of this threonine greatly affects cellular localization and transcriptional activity of this Smad [41, 42]. TAK1 kinase activity is essential for this interaction of TAK1 with R-Smads [42]. As a consequence of this kinase dependency, it has been reported that the use of (5Z)-7-Oxozeaenol results in similar effects on C-terminal Smad1/5 and Smad2/3 phosphorylation as knockout of TAK1 [41]. We did not have access to cartilage-specific TAK1 knockout animals, but with the use of (5Z)-7-Oxozeaenol, we show for the first time that this selective TAK1 kinase inhibitor greatly affects TGFβ1-induced C-terminal R-Smad phosphorylation both in vitro and ex vivo in articular cartilage.

A limitation of our study is the possibly imperfect specificity of the used compounds, making it possible that off-target effects can explain our observations and interfere with our conclusions. However, in the experiments with the caALKs, we did not observe a significant effect of SB-505124 or (5Z)-7-Oxozeaenol on caALK1-induced pSmad1/5, or an effect of (5Z)-7-Oxozeaenol on caALK5-induced pSmad2. Therefore we do not think that these compounds have off-target effects on ALK1. Furthermore, a dose-response curve showed that SB-505124 inhibited TGFβ-induced Smad1/5 phosphorylation more potently than Smad2/3 phosphorylation, demonstrating that this effect of SB-505124 on TGFβ signaling is not an off-target effect obtained at high dosage, and this compound therefore cannot be used to specifically inhibit TGFβ-induced Smad2 in chondrocytes.

Another limitation of our study is that we did not include in vivo data. Unfortunately, the in vivo use of both SB-505124 and LDN-193189 is difficult because of their unfavorable pharmacokinetic properties, resulting in the need for daily re-administation via injection [33]. Up to now, only one study has used SB-505124 as a TGFβ blocker in vivo in a model of OA; i.e. in anterior cruciate ligament transection in mice [46]. In this study, inhibition of TGFβ signaling with 1 mg/kg/day of SB-505124 attenuated OA development, confirming that inhibition of TGFβ signaling with a small molecule inhibitor is a feasible and promising approach to treat OA. However, the use of a higher dose of SB-505124 (2.5 mg/kg/day) was detrimental for cartilage because this resulted in proteoglycan depletion, indicating that total TGFβ inhibition comes with a risk. Possibly, a small molecule inhibitor that prevents TGFβ-induced pSmad1/5 but leaves pSmad2 unaffected will not have this risk. To our knowledge, both LDN-193189 and (5Z)-7-Oxozeaenol have not been used in vivo to study their effects on OA development. They have been used in other applications, e.g. LDN-193189 in atherosclerosis research and (5Z)-7-Oxozeaenol in cancer studies; however, this was not to specifically modulate TGFβ signaling but to inhibit BMP and TAK1 signaling, respectively. Therefore it is as yet unknown how these inhibitors modulate TGFβ signaling in vivo or in OA.

## Conclusions

In conclusion, with the use of the small molecule inhibitors SB-505124 and LDN-193189 we were unable to direct TGFβ1-signaling towards either Smad1/5 or Smad2/3 phosphorylation in cartilage. Our data suggest that in cartilage, ALK5 plays a central role in both TGFβ-induced Smad1/5 and Smad2/3 phosphorylation, making it difficult to separate the Smad pathways with the use of currently available intracellular small molecule inhibitors of the ALK receptors. Furthermore, we showed that treatment with the TAK1 inhibitor (5Z)-7-Oxozeaenol inhibits TGFβ-induced C-terminal phosphorylation of both Smad1/5 and Smad2/3, suggesting a link between the non-canonical and the canonical TGFβ pathway in cartilage.

## Additional files

Additional file 1: Figure S1. Expression of ALK1, ALK2, ALK3 and ALK5 mRNA in primary bovine cartilage and chondrocytes. **a** With the use of qPCR, expression of ALK1, ALK2, ALK3 and ALK5 was measured in both freshly isolated cartilage explants and in primary chondrocytes after 1 week of cell culture in DMEM/F12 supplemented with 10% non-heat-inactivated FCS without passage. All four ALKs were readily detected in both groups, but expression of all the receptors was higher in freshly isolated tissue. For calculations of the -ΔCt, two reference genes were used: *bGapdh* and *bRps14*. (PDF 2065 kb)

Additional file 2: Figure S3. Quantification of TGFβ-induced pSmad2 and pSmad1/5 in cartilage explants. Quantification of the western blot as shown in Fig. 4a. The experiment was repeated three times. Significance was not obtained due to variation between experiments. pSmad levels were normalized to vinculin levels and plotted as a relative amount in arbitrary units (AU) compared to the control group. (PDF 2278 kb)

Additional file 3: Figure S2. LDN-193189 in a concentration of 0.05 μM inhibits basal *ID1* expression in cartilage explants. Primary chondrocytes were incubated with LDN-193189 for 2 h in a dose of 0.05 μM and *bId1* expression was measured using qPCR. LDN-193189 significantly inhibited *bId1* expression showing the bioactivity of this compound in cartilage explants. (PDF 2122 kb)

## Abbreviations

μl: Microliter
μM: Micromolar (10^-6^ mol per liter)
ALK: Activin receptor-like kinase
BCA: Bicinchoninic acid assay
BMP: Bone morphogenetic protein
caALK: Constitutively active activin receptor-like kinase
CDK: Cyclin-depdendent kinase
cDNA: Complementary deoxyribonucleic acid
DMEM: Dulbecco’s modified Eagle’s medium
DNAse: Deoxyribonuclease
dNTP: Deoxynucleoside triphosphate
dT: Deoxythymidine
DTT: Dithiothreitol
EDTA: Ethylenediaminetetraacetic acid
ERK: Extracellular signal-regulated kinase, also known as mitogen-activated protein kinase (MAPK1)
*g*: Gravitational force
Gapdh: Glyceraldehyde-3-phosphate dehydrogenase
h: Hour
HRP: Horseradish peroxidase
IC_50_: Half maximal inhibitory concentration
JNK: JUN N-terminal kinase
kDa: Kilodalton
LacZ: β-galactosidase
m/v: Mass per volume
mAb: monoclonal antibody
MAPK: Mitogen-activated protein kinase
min: Minute
ml: Milliliter
mm: Millimeter
M-MLV: Moloney murine leukemia virus
MMP: Matrix metalloproteinase
(m)RNA: (messenger) Ribonucleic acid
Ng: Nanogram
OA: Osteoarthritis
PCR: Polymerase chain reaction
R-Smad: receptor-regulated Smad
TAK1: TGFβ-activated kinase 1, also known as: mitogen-activated protein kinase kinase kinase 7 (MAP3K7)
TGFβ: Transforming growth factor beta
U: units
