# Supplementary figures and images for: TGFβ1-induced SMAD2/3 and SMAD1/5 phosphorylation are both ALK5-kinase-dependent in primary chondrocytes and mediated by TAK1 kinase activity
Source: Arthritis Res Ther. 2017 May 31;19:112. doi: 10.1186/s13075-017-1302-4 (PMC5452635; doi:10.1186/s13075-017-1302-4)

Figure S1

ALK expression in bovine chondrocytes and cartilage

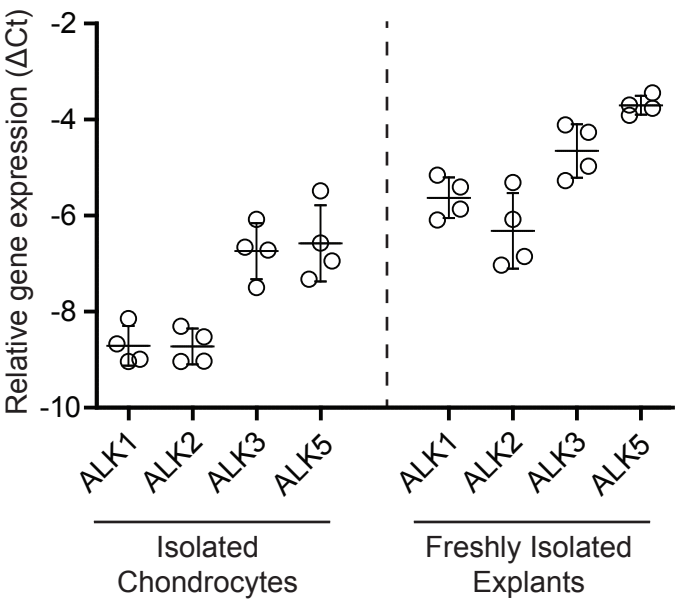

Supplement: Supplementary file 1 — Expression of ALK1, ALK2, ALK3 and ALK5 mRNA in primary bovine cartilage and chondrocytes. a With the use of qPCR, expression of ALK1, ALK2, ALK3 and ALK5 was measured in both freshly isolated cartilage explants and in primary chondrocytes after 1 week of cell culture in DMEM/F12 supplemented with 10% non-heat-inactivated FCS without passage. All four ALKs were readily detected in both groups, but expression of all the receptors was higher in freshly isolated tissue. For calculations of the -ΔCt, two reference genes were used: bGapdh and bRps14. (PDF 2065 kb) [file 13075_2017_1302_MOESM1_ESM.pdf]

Figure S3

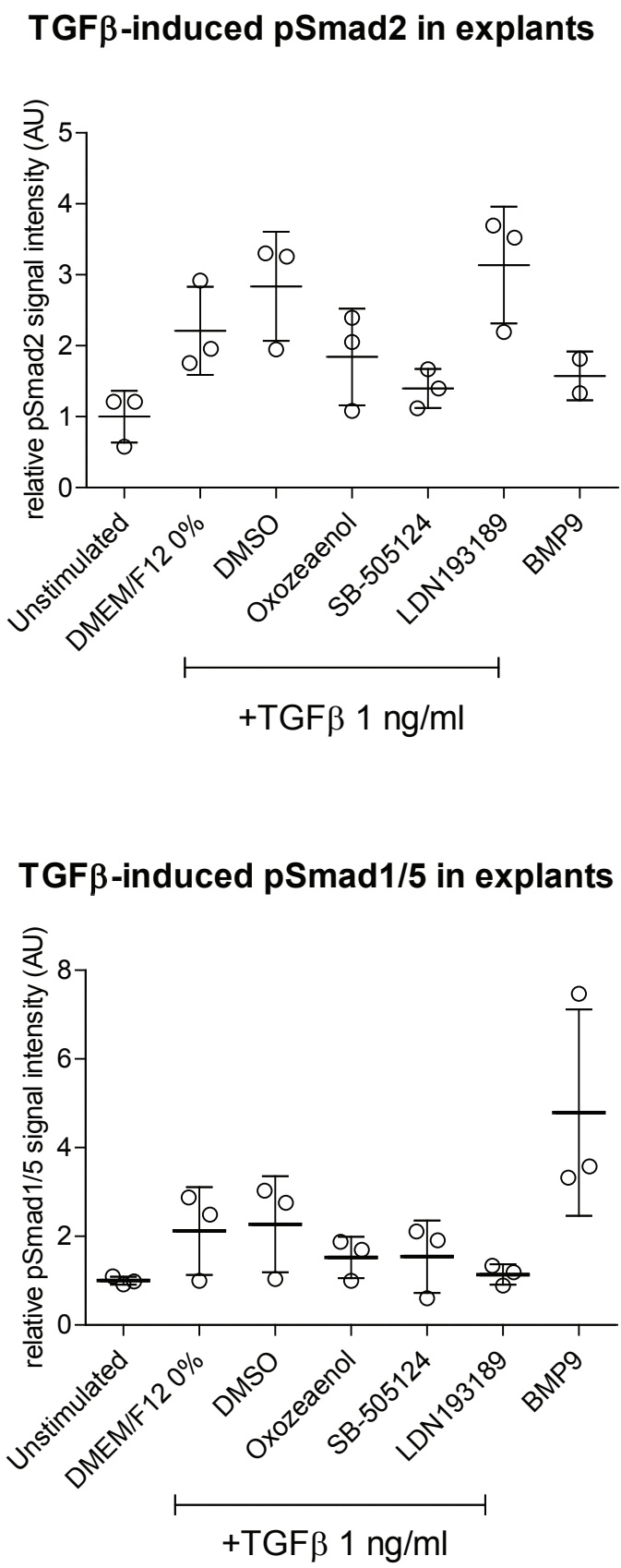

Supplement: Supplementary file 2 — Quantification of TGFβ-induced pSmad2 and pSmad1/5 in cartilage explants. Quantification of the western blot as shown in Fig. 4a. The experiment was repeated three times. Significance was not obtained due to variation between experiments. pSmad levels were normalized to vinculin levels and plotted as a relative amount in arbitrary units (AU) compared to the control group. (PDF 2278 kb) [file 13075_2017_1302_MOESM2_ESM.pdf]

Figure S2

ID1 expression in cartilage explants

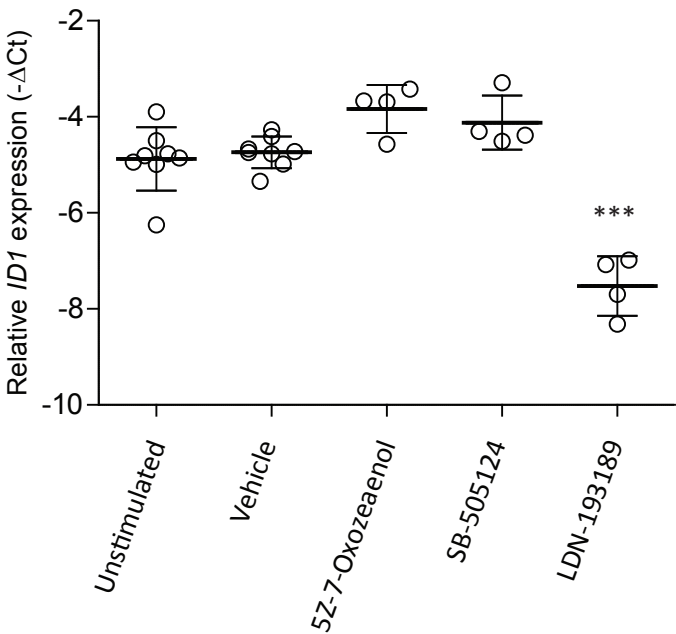

Supplement: Supplementary file 3 — LDN-193189 in a concentration of 0.05 μM inhibits basal ID1 expression in cartilage explants. Primary chondrocytes were incubated with LDN-193189 for 2 h in a dose of 0.05 μM and bId1 expression was measured using qPCR. LDN-193189 significantly inhibited bId1 expression showing the bioactivity of this compound in cartilage explants. (PDF 2122 kb) [file 13075_2017_1302_MOESM3_ESM.pdf]
